# Elephantiasis scrotal revelant un cancer de la prostate

**DOI:** 10.11604/pamj.2014.17.190.3601

**Published:** 2014-03-12

**Authors:** Amal Taghy, Badreddine Hassam

**Affiliations:** 1 Service de Dermatologie-Vénérologie, CHU Ibn Sina, Maroc; 2 Faculté de Médecine et de Pharmacie, Med V Souissi Rabat, Maroc

**Keywords:** Eléphantiasis, scrotum, organes génitaux, cancer de la prostate, elephantiasis, scrotum, genitals, prostate cancer

## Image en medicine

L’éléphantiasis scrotal(LO) est une augmentation considérable du volume des bourses s'observant surtout en zones d'endémie filarienne mais pouvant être d'origine primitive ou secondaire à la destruction de ganglions lymphatiques pelviens et/ou inguinaux ou à une compression par certains cancers génitaux ou lymphomes. Les complications induites sont fonctionnelles, esthétiques et infectieuses retentissant sur la qualité de vie et les problèmes sexuels sont difficiles à étudier en raison de la maladie causale qui peut elle-même en engendrer. Le traitement est difficile et repose sur la chirurgie réparatrice et le traitement de la cause sous-jacente. Nous rapportant un cas d'un homme de 52 ans, sans antécédents, consultant pour un lymphoedème génital évoluant depuis deux ans. L'examen clinique objectivait une peau scrotale épaissie et scléreuse, des lésions papulo-kératosiques diffuses au niveau du scrotum et de la verge, des difficultés mictionnelles et une impuissance sexuelle. Un bilan paranéoplasique était effectué mettant en évidence un cancer de la prostate au stade T2a, N2, Mo. Le score de Gleason était à (3 + 2 = 5) et la PSA à 12ng/ml. Le patient a été transféré au service d'urologie où une prostatectomie radicale était réalisée. Un traitement chirurgical était effectué ultérieurement consistant en une exérèse-plastie en Z en un temps, un drainage par lame maintenue trois jours au niveau du scrotum et une plastie pénienne.

**Figure 1:**
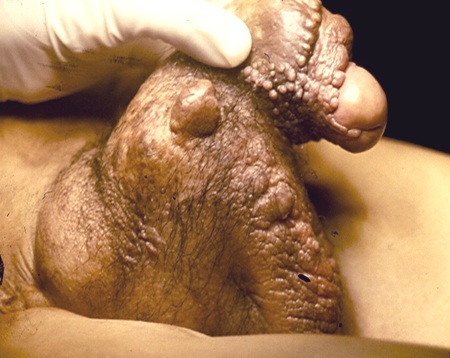
Eléphantiasis génital avec papules kératosiques couleur chaire, de tailles variables, confluentes par endroits et siégeant de façon diffuse au niveau du scrotum et de la verge

